# Supplementary material for: Up and Down γ-Synuclein Transcription in Dopamine Neurons Translates into Changes in Dopamine Neurotransmission and Behavioral Performance in Mice
Source: Int J Mol Sci. 2022 Feb 4;23(3):1807. doi: 10.3390/ijms23031807 (PMC8836558; doi:10.3390/ijms23031807)
Supplement: Supplementary file 1 [file ijms-23-01807-s001.zip › ijms-1571784-supplementary.pdf]

International Journal of *Molecular Sciences*

MDPI

Supplementary Materials

Up-and-down γ-synuclein transcription in dopamine neurons translates into changes in dopamine neurotransmission and behavioral performance in mice

Rubén Pavia-Collado [1,2,3,4], Raquel Rodríguez-Aller [5,6], Diana Alarcón-Arís [1,2,3], Lluís Miquel-Rio [1,2,3], Esther Ruiz-Bronchal [1,2,3], Verónica Paz [1,2,3], Leticia Campa [1,2,3], Mireia Galofré [2,7,8], Véronique Sgambato [9], Analia Bortolozzi [1,2,3*]

[1] Institut d'Investigacions Biomèdiques de Barcelona (IIBB), Spanish National Research Council (CSIC), 08036 Barcelona, Spain; ruben.pavia@iibb.csic.es (R.P.-C.); dalarcar5@gmail.com (D.A.-A.); lluis.miquel@iibb.csic.es (L.M.-R.); esther.ruiz@iibb.csic.es (E.R.-B.); veronica.paz@iibb.csic.es (V.P.); leticia.campa@iibb.csic.es (L.C.); analia.bortolozzi@iibb.csic.es (A.B.)

[2] Institut d'Investigacions Biomèdiques August Pi i Sunyer (IDIBAPS), 08036 Barcelona, Spain

[3] Centro de Investigación Biomédica en Red de Salud Mental (CIBERSAM), ISCIII, 28029 Madrid, Spain

[4] miCure Therapeutics LTD., Tel-Aviv, 6423902 Israel

[5] CHU de Quebec Research Center, Axe Neurosciences. Department of Molecular Medicine, Faculty of Medicine, Université Laval, Quebec City, QC G1V 4G2, Canada; raquel.rodriguez-aller.1@ulaval.ca (R.R.-A.)

[6] CERVO Brain Research Centre, Quebec City, QC G1J 2G3, Canada

[7] Laboratory of Stem Cells and Regenerative Medicine, Department of Biomedicine, Faculty of Medicine and Health Science, University of Barcelona, 08036 Barcelona, Spain; mireiagalofre@ub.edu (M.G.)

[8] Centro de Investigación Biomédica en Red de Enfermedades Neurodegenerativas (CIBERNED), ISCIII, 28029 Madrid, Spain

[9] Université de Lyon, CNRS UMR 5229, Institut des Sciences Cognitives Marc Jeannerod, Bron, France; veronique.sgambato@inserm.fr (V.S.)

* Correspondence: analia.bortolozzi@iibb.csic.es; Tel.: +34 93 363 8313

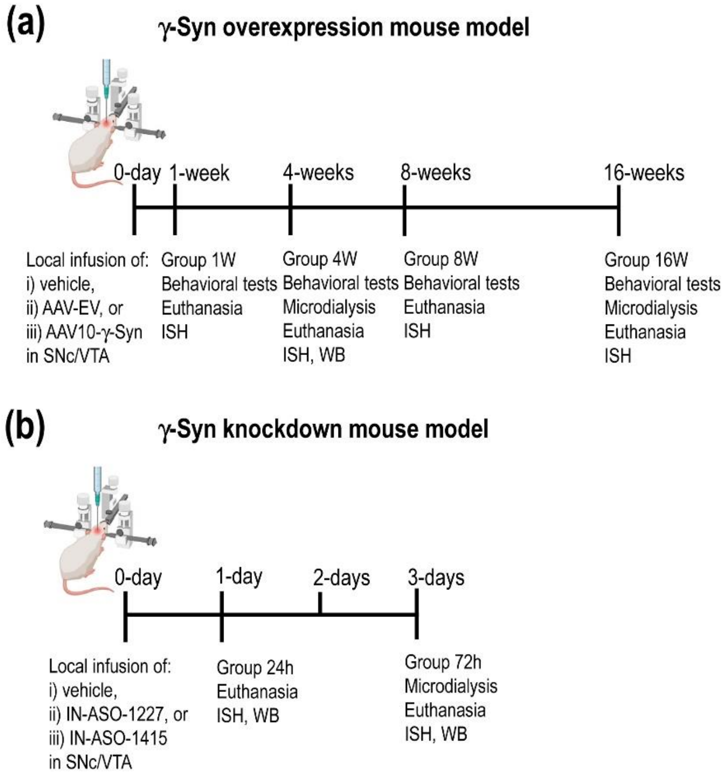

Supplemental Figure S1. Treatment timeline. **(a)** γ-Syn overexpression mouse model. Mice received unilaterally: i) vehicle, ii) empty vector with noncoding stuffer DNA (AAV-EV, 1 µl, 2,73 x 10^{13} gc/mL), or iii) AAV10 vector containing a cytomegalovirus promoter to drive the expression of γ-Syn (AAV10- γ-Syn, 1 µl, 1.34 x 10^{13} gc/mL) into SNc/VTA. Different experimental procedures were carried out at 1, 4, 8 and 16-weeks (W) post-injection. **(b)** γ-Syn knockdown mouse model. Mice received a single acute administration of: i) vehicle, ii) IN-conjugated nonsense ASO sequence (IN-ASO-1227, 1 µl, 60 µg), or iii) IN-ASO-1415 targeting γ-Syn (1 µl, 60 µg) into SNc/VTA. Different experimental procedures were carried out at 1 and 3-days post-infusion. Abbreviations: ISH, in situ hybridization; WB, Western blot.

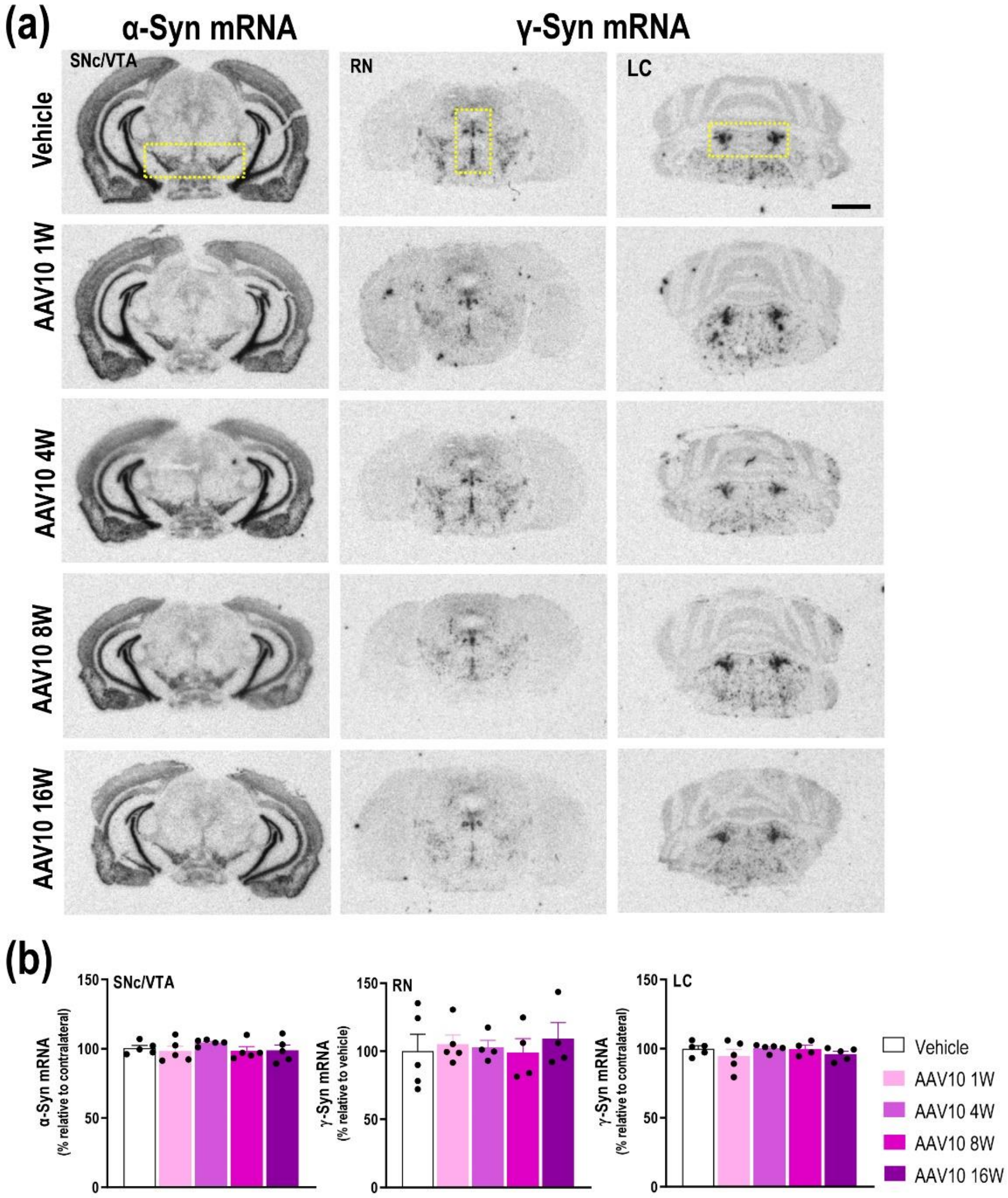

Supplemental Figure S2. α- and γ-Syn mRNA expression profile in midbrain monoaminergic brain areas of mice overexpressing γ-Syn in SNc/VTA. Mice received unilaterally 1 µl of AAV10 vector containing a cytomegalovirus promoter to drive the expression of γ-Syn or vehicle into SNc/VTA and euthanized at 1, 4, 8 and 16-weeks (W) post-injection (see Figure 1). **(a)** Coronal brain sections showing α-Syn mRNA levels in SNc/VTA and γ-Syn mRNA levels in the RN and LC assessed by in situ hybridization. Yellow frames indicate the brain regions quantified in b. Scale bar: 500 µm. **(b)** No differences between groups were detected for α- and γ-Syn RNA expression in the brain areas analyzed. Data are represented as the mean ± SEM (n=5 mice/group, same mice used in Figure 1). Abbreviations: SNc, substantia nigra compacta; VTA, ventral tegmental area; RN, raphe nuclei; LC, locus coeruleus.

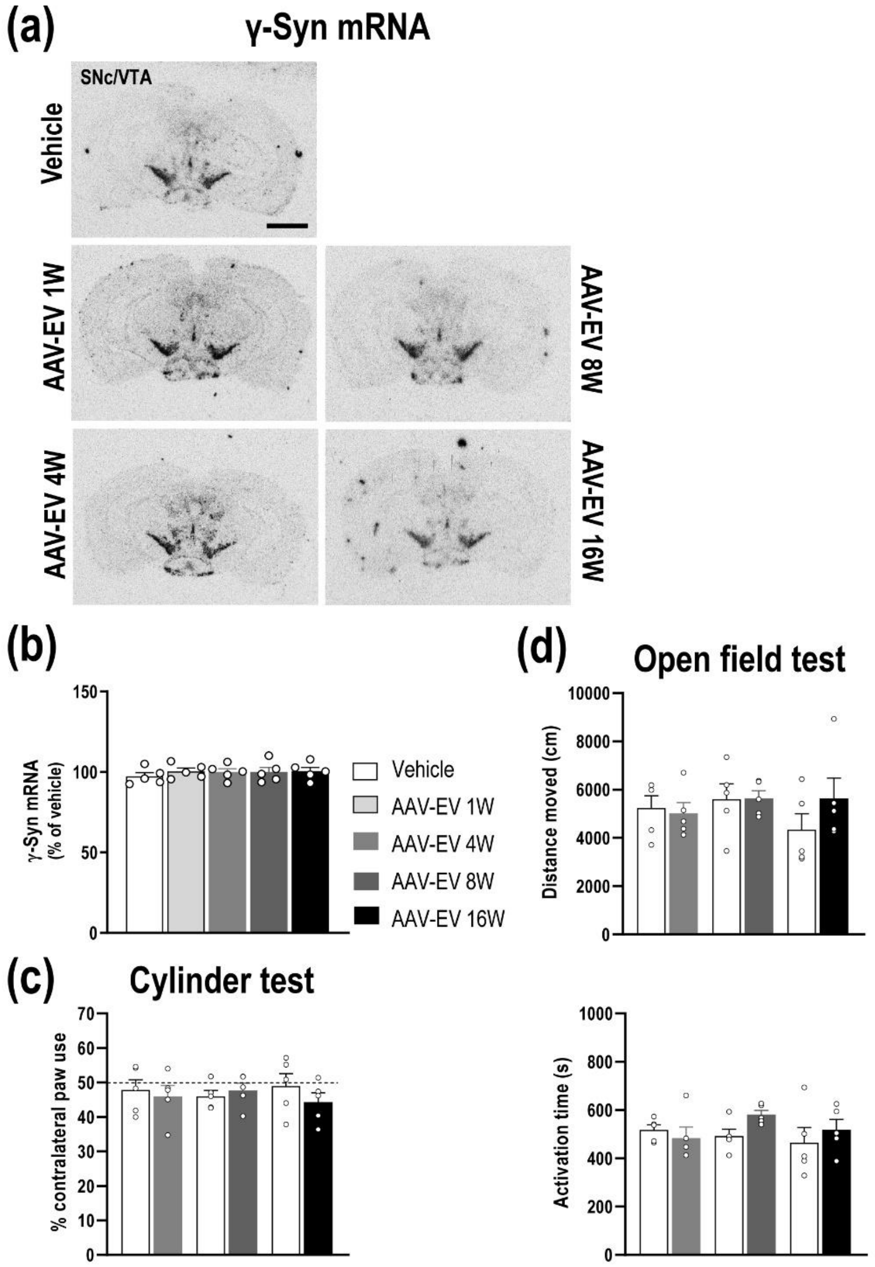

Supplemental Figure S3. AAV-empty vector (AAV-EV) injection did not modify mouse γ-Syn mRNA expression, nor did it affect behavioral performance. Mice received unilaterally 1 µl of AAV-EV or vehicle into SNc/VTA and euthanized at 1, 4, 8 and 16-weeks (W) post-injection (see Figure 1). **(a)** Coronal brain sections showing γ-Syn mRNA levels in SNc/VTA assessed by *in situ* hybridization. Scale bar: 500 µm. **(b)** Analysis of SNc/VTA autoradiograms did not show statistical differences between experimental groups (one-way ANOVA). **(c,d)** Mice injected with vehicle or AAV-EV showed comparable performance in the cylinder test (c) and open field test (two-way ANOVA). Data are represented as the mean ± SEM (n=5 mice/group). Abbreviations: SNc, substantia nigra compacta; VTA, ventral tegmental area.

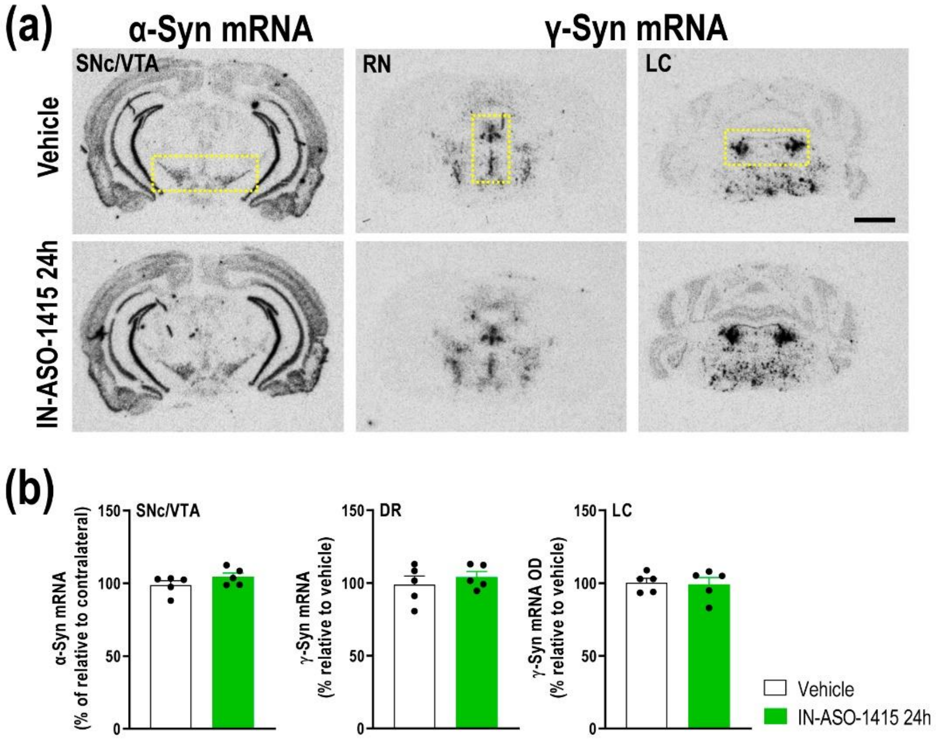

Supplemental Figure S4. α- and γ-Syn mRNA expression profile in midbrain monoaminergic brain areas of IN-ASO-1415-induced γ-Syn knockdown mice. Mice received unilaterally 1 µl of IN-ASO-1415 (total dose of 60 µg) or vehicle into SNc/VTA and euthanized at 24 h post-infusion (see Figure 5). **(a)** Coronal brain sections showing α-Syn mRNA levels in SNc/VTA and γ-Syn mRNA levels in the RN and LC assessed by *in situ* hybridization. Yellow frames indicate the brain regions quantified in b. Scale bar: 500 µm. **(b)** No differences between groups were detected for α- and γ-Syn RNA expression in the brain areas analyzed. Data are represented as the mean ± SEM (n=5 mice/group, same mice used in Figure 5). Abbreviations: SNc, substantia nigra compacta; VTA, ventral tegmental area; RN, raphe nuclei; LC, locus coeruleus.
